# MIF Participates in *Toxoplasma gondii*-Induced Pathology Following Oral Infection

**DOI:** 10.1371/journal.pone.0025259

**Published:** 2011-09-22

**Authors:** Marta G. Cavalcanti, Jacilene S. Mesquita, Kalil Madi, Daniel F. Feijó, Iranaia Assunção-Miranda, Heitor S. P. Souza, Marcelo T. Bozza

**Affiliations:** 1 Departamento de Imunologia, Instituto de Microbiologia Professor Paulo de Góes, Universidade Federal do Rio de Janeiro (UFRJ), Rio de Janeiro, Brazil; 2 Departamento de Virologia, Instituto de Microbiologia Professor Paulo de Góes, Universidade Federal do Rio de Janeiro (UFRJ), Rio de Janeiro, Brazil; 3 Serviço de Doenças Infecciosas e Parasitárias, Universidade Federal do Rio de Janeiro (UFRJ), Rio de Janeiro, Brazil; 4 Departamento de Clínica Médica, Laboratório Multidisciplinar de Pesquisa, Universidade Federal do Rio de Janeiro (UFRJ), Rio de Janeiro, Brazil; 5 Departamento de Patologia, Hospital Universitário Clementino Fraga Filho, Universidade Federal do Rio de Janeiro (UFRJ), Rio de Janeiro, Brazil; 6 Laboratório Sérgio Franco, Rio de Janeiro, Brazil; McGill University, Canada

## Abstract

**Background:**

Macrophage migration inhibitory factor (MIF) is essential for controlling parasite burden and survival in a model of systemic *Toxoplasma gondii* infection. Peroral *T. gondii* infection induces small intestine necrosis and death in susceptible hosts, and in many aspects resembles inflammatory bowel disease (IBD). Considering the critical role of MIF in the pathogenesis of IBD, we hypothesized that MIF participates in the inflammatory response induced by oral infection with *T. gondii*.

**Methodology/Principal Findings:**

*Mif* deficient (*Mif^−^/^−^*) and wild-type mice in the C57Bl/6 background were orally infected with *T. gondii* strain ME49. *Mif^−^/^−^* mice had reduced lethality, ileal inflammation and tissue damage despite of an increased intestinal parasite load compared to wt mice. Lack of MIF caused a reduction of TNF-α, IL-12, IFN-γ and IL-23 and an increased expression of IL-22 in ileal mucosa. Moreover, suppressed pro-inflammatory responses at the ileal mucosa observed in *Mif^−^/^−^* mice was not due to upregulation of IL-4, IL-10 or TGF-β. MIF also affected the expression of matrix metalloproteinase-9 (MMP-9) but not MMP-2 in the intestine of infected mice. Signs of systemic inflammation including the increased concentrations of inflammatory cytokines in the plasma and liver damage were less pronounced in *Mif^−^/^−^* mice compared to wild-type mice.

**Conclusion/Significance:**

In conclusion, our data suggested that in susceptible hosts MIF controls *T. gondii* infection with the cost of increasing local and systemic inflammation, tissue damage and death.

## Introduction

The immune/inflammatory response to *Toxoplasma gondii* infection is essential to control parasite replication and tissue spread but also can cause tissue damage, being descisive to pathogenesis. Selective tissue invasion by *T. gondii* causes compartmental immune responses unique to each tissue such as the one present in the placenta, central nervous system and intestinal mucosa, while parasite spreading promotes a systemic response that resembles bacterial sepsis [1]–[3]. Inhibition or abrogation of TNF-α, IL-12 and/or IFN-γ increase host susceptibility to toxoplasmosis as a result of uncontrolled parasite burden [4]–[6]. In natural *T. gondii* infection through the oral route, exacerbated inflammatory responses at the small intestine (ileitis) resemble human inflammatory bowel disease (IBD) [1], [7].The inflammatory response coordinate by IL-23/IL-22 is involved in *T. gondii*-induced disruption of intestinal homeostasis and immunopathology, an effect at least in part due to increase expression of matrix metalloproteinase-2 (MMP) [7]. These pro-inflammatory mediators and MMPs are involved in the immune pathogenesis and tissue damage in *T. gondii* peoral infection, increasing host morbidity and mortality despite parasite control [7]–[9]. Deficient regulation of the inflammatory response observed in the absence of IL-10 results in massive leukocyte infiltration, extensive tissue damage despite control of parasite load, systemic inflammation and increase lethality [2].

MIF is a versatile molecule that comprises hormonal and enzymatic activities in addition to exert pro-inflammatory and chemotactic functions [10]–[13]. First described as a T-cell-derived factor involved in the inhibition of macrophage migration in delayed–type hypersensitivity response, MIF is also produced by other immune cells such as macrophages, neutrophils and eosinophils but also non immune cells such as endothelial, epithelial, muscle and pituitary cells [10]–[15]. MIF expression and production are regulated by adrenocorticotrophic hormone which results in counter-regulation of cortisol [11], [16]. MIF production is also regulated by TNF and LPS-Toll-like receptor 4 (TLR4) in a positive feedback cascade resulting in enhanced proinflammatory responses [15], [17], [18]. Furthermore, MIF is an essential regulator of innate and adaptive immune responses with a crucial role in host protection against different pathogens and is involved in the pathogenesis of a broad spectrum of infectious and non infectious inflammatory diseases [10]–[23].

In parasitic infections MIF is involved both in protective as well as immune pathogenic responses [12], [21], [24]–[28]. Upon systemic infection with *T. gondii*, IFN-γ and TNF-α mediated responses are upregulated by MIF, and *Mif^−^/^−^* mice are more susceptible to infection presenting increased parasite burden [27]. A recent study also demonstrated an increased susceptibility, reduced IL-12 production and dendritic cell activation on *Mif^−^/^−^* mice in the Balb/c background to an oral *T. gondii* infection [28]. Balb/c mice are naturally resistant to oral infection with *T. gondii*, while C57BL/6 are highly susceptible and display intestinal inflammation, especially in the ileum [29]–[31].

Considering the role of MIF on intestinal inflammation, we hypothesize that MIF participates in the pathogenesis of *T. gondii*-induced intestinal tissue damage. Our results indicate that MIF mediates pathogenic responses during *T. gondii* oral infection by exacerbating compartmental and systemic inflammatory responses. The lack of MIF caused an overall reduction of tissue damage despite of an increase in local parasite burden.

## Materials and Methods

### Mice

C57BL/6 (wild type, wt) and *Mif^−^/^−^* (backcrossed to the C57BL/6 genetic background, n = 8) were obtained from the animal facility at the Universidade Federal do Rio de Janeiro (UFRJ, Rio de Janeiro). Sex and age-matched (8–12 week-old) C57BL/6 were used as controls for *Mif^−^/^−^*. The animals were kept at constant temperature (25°C) with free access to chow and water in a room with a 12 h light/dark cycle. All animal experiments were performed in accordance to Brazilian Government's ethical and animal experiment regulations. The experiments were approved by the Institutional Animal Welfare Committee (approval ID: CEUA/CCS/UFRJ/IMPPG 011).

### Parasites and per oral infection

The *T. gondii* strain ME49 was kindly provided by Dr R. Gazzinelli, (UFMG, Minas Gerais, Brazil) and Dr J. Lannes-Vieira (FIOCRUZ, Rio de Janeiro, Brazil). For peroral infection, mice were infected by gavage using 20 and 100 ME49 cysts/animal. Cysts were obtained from C57BL/6 brain homogenates as previously described [2].

### Detection of *T. gondii* DNA

Parasites in tissues were quantified by a real-time polymerase chain reaction (RT-PCR) as previously described [7] targeting a repetitive 529-bp DNA fragment of *T. gondii* (GenBank accession no AF487550) [32] which are specific for a cryptic gene as described [33]. Fifty to 100 µg of ileum and liver tissues were crushed in a rotor-stator after frozen in liquid nitrogen and total DNA were isolated with DNAzol reagent (Invitogen Life Technologies) following the manufactures protocol. Five hundred ng of total DNA extracted from each sample were submitted to real-time PCR using Power SYBR® Green PCR master mix (Applied Biosystems) using each oligonucleotide primer (TOX-9, 5′-AGGAGAGATATCAGGACTGTAG-3′; TOX-10, 5′-GCGTCGTCTCGTCTAGATCG-3′). The amplification was performed using one cycle at 95°C for 10 min, 45 cycles at 95°C for 30 s, 60°C for 1 min. A standard curve was performed using 5000-5 pg *T. gondii* DNA to quantification of total DNA of samples.

### Determination of small intestinal length, histopathology and histological scores

Small intestinal shortening was determined by measuring whole intestinal segment of wt and *Mif^−^/^−^* controls and *T. gondii*-infected mice. It was calculated by dividing the difference of the mean length of the small intestine from control mice minus the length from infected mice at day 8 after infection and then multiplied by 100 over the mean length of mice small intestines [34]. Intestines, liver and brain were fixed in 10% neutral buffered formalin, embedded in paraffin, sectioned at 5 µm, and stained with hematoxylin and eosin using standard techniques. Blinded samples were submitted for semi-quantitative histopathologic analysis. Histological scores were determined in fixed and paraffin-embedded tissue sections taken from the terminal ileum, as previously described [34]. Liver sections were analyzed for the numbers of inflammatory foci at a magnification of ×200 at day 9 after infection in each group. We analyzed the number of inflammatory foci per field (6.5×9 mm, microscope Zeiss, Germany) counting 10 fields of each section.

### Determination of cytokine concentrations at the intestinal tissue and plasma

Plasma samples were obtained to measure cytokine concentrations. IFN-γ and TNF-α concentrations were measured by a commercial ELISA from Peprotech (NJ, USA). Concentrations of IL-10 was determined by ELISA using a commercial kit from R&D Systems (MN, USA).

### RT-PCR analysis of matrix metalloproteinases and cytokines

Approximately 50–100 µg of ileum tissue were crushed in a rotor–stator after frozen in liquid nitrogen and total RNA were isolated with TRIzol reagent (Invitogen Life Technologies) following the manufactures protocol. Four micrograms of total RNA extracted were reverse transcribed using a High capacity cDNA reverse transcription kit (Applied Biosystems, Foster City, CA). Each sample was submitted to real-time PCR using Power SYBR® Green PCR master mix (Applied Biosystems). The reactions were carried out using specific primers for the following genes: MMP-2 (forward, 5′-CAATCTTTTCTGGGAGCTC-3′; reverse, 5′-GCTGATACTGACACTGGTACTG-3′), MMP-9 (forward, 5′-CCTGGAACTCACACGACATCTTC-3′; reverse, 5′-TGGAAACTCACACGCCAGAA-3′), TNF-α (forward, 5′-CCTCACACTCAGATCATCTTCTCA-3′; reverse, 5′- TGGTTGTCTTTGAGATCCATGC-3′), IL-6 (forward, 5′- TCATATCTTCAACCAAGAGGTA-3′; reverse, 5′- CAGTGAGGAATGTCCACAAACTG-3′), IFN-γ (forward, 5′-AGCAACAGCAAGGCGAAAA-3′; reverse, 5′- CTGGACCTGTGGGTTGTTGA -3′), IL-17A (forward, 5′-TCCCTCTGTGATCTGGGAAG-3′; reverse, 5′-CTCGACCCTGAAAGTGAAGG-3′), TGF-β (forward, 5′-GACCGCAACAACGCCATCTA-3′; reverse, 5′-AGCCCTGTATTCCGTCTCCTT-3′), IL-12p35 (forward, 5′-CCACCCTTGCCCTCCTAAAC-3′; reverse, 5′-GGCAGCTCCCTCTTGTTGTG-3′), IL-23 (forward, 5′-TGGCATCGAGAAACTGTGAGA-3′; reverse, 5′-TCAGTTCGTATTGGTAGTCCTGTTA-3′), IL-22 (forward, 5′-GACAGGTTCCAGCCCTACAT-3′; reverse, 5′-GTCGTCACCGCTGATGTG-3′), IL-10 (forward, 5′-TAAGGGTTACTTGGGTTGCCAAG-3′; reverse, 5′-CAAATGCTCCTTGATTTCTGGGC-3′). The samples were subjected to 45 amplification cycles consisting in 95°C for 30 s, 60°C for 1 min. The expression of the hypoxanthine guanine phosphoribosyl transferase 1 (Hprt1) gene (forward, 5′-GCTGGTGAAAAGGACCTCT-3′; reverse 5′-CAC AGG ACT AGA ACA CCT GC-3′) was used to normalize the results, which were presented as fold induction of mRNA expression relative to control samples. Moreover, mouse TaqMan pre-developed assay reagents (Applied Biosystems) were used for IL-4 and Hprt amplification. The analyses of relative gene expression data were performed by 2^−ΔΔC^_T_ method [35].

### Statistical analysis

Data are expressed as means ± SEM. The statistical significance of differences in mean values was determined by using Student's t test or ANOVA. Survival data are presented as a Kaplan-Meier survival curve and analyzed with Log-rank test by software Prism 5 (USA). Differences of at least p≤0.05 are considered significant.

## Results

### Reduced mortality and morbidity of *Mif^−/−^* mice after *T. gondii* oral infection

**Figure 1:**
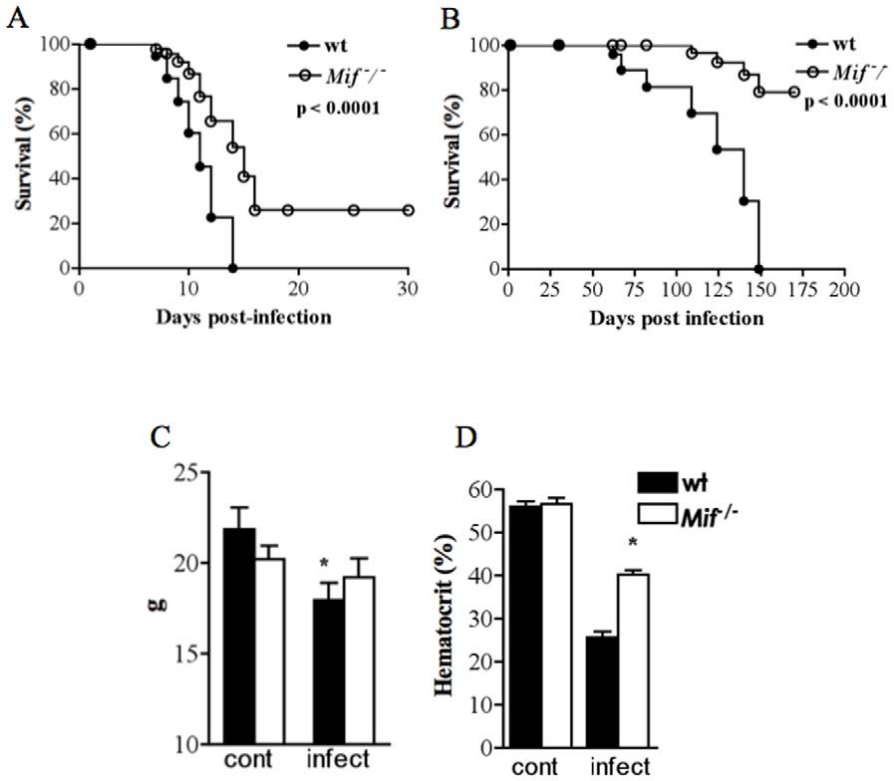
MIF increases morbidity and mortality during peroral *T. gondii* infection. Survival curves of *Mif* ^−^/^−^ (C57BL/6) mice (6–8 weeks old) and age and sex-matched wild type controls orally infected with 100 (A) and 20 cysts (B) of ME-49 strain of *T. gondii*. Morbidity was evaluated by determining animal (C) relative weight loss and (D) hematocrit. One representative experiment out of three performed. Data from 8 to 10 animals per group are given as mean ± SEM, and p values were determined by t-test (* p≤0.05) or by Log-rank test (A, B). Cont, control not infected.

It has been recently demonstrated that MIF determines host susceptibility to *T. gondii* infection by peritoneal route [27]. *Mif^−^/^−^* mice presente increased parasite load and systemic inflammatory response when compared to wt mice. However, *T. gondii* infection through the oral route might determine a different outcome depending on the inflammatory response within the intestinal mucosa [36], . To define the role of MIF during natural *T. gondii* infection in susceptible hosts, wt and *Mif^−^/^−^* mice were orally infected with 100 or 20 cysts per animal. Wt mice infected with 100 cysts were all dead after 10–12 days post infection while *Mif^−^/^−^* mice had a significant increase of survival (Fig. 1A). The increase survival of *Mif^−^/^−^* compared to wt mice was even more pronounced upon infection with 20 cysts/animal (Fig. 1B). To assess morbidity during acute *T. gondii* infection, we used weight loss and hematocrit measurement, considered good markers of morbidity in acute acquired toxoplasmosis [38]. Although both wt and *Mif^−^/^−^* mice presented weight loss, it was more severe in wt than on *Mif^−^/^−^* mice (Fig. 1C). Similarly, hematocrit was significantly reduced in infected wt when compared to *Mif^−^/^−^* mice (Fig. 1D). Thus, *Mif^−^/^−^* mice displayed increased resistance to acute infection and reduced morbidity following per oral *T. gondii* infection.

### Reduced ileal damage despite of increased parasite loads in the absence of MIF

**Figure 2:**
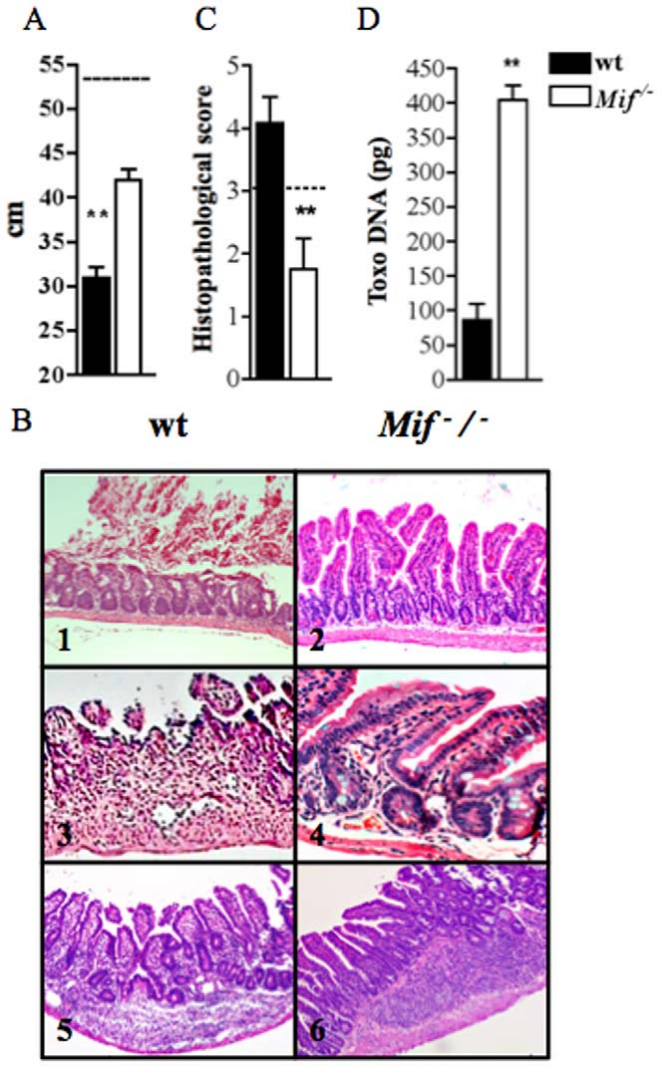
*T. gondii*-induced ileitis is mediated by MIF that induces severe tissue damage despite efficient parasite control. (A) Shortening of small intestine of wt and *Mif* ^−^/^−^ mice (n = 6) at 9 days post-infection (dpi). (B) Histopathologycal analysis by hematoxilin and eosin (HE) of terminal ileum was performed at 9 dpi in wt and *Mif* ^−^/^−^. Magnification are ×200 (B1, 2, 5 and 6) and ×400 (B3 and 4). In (C) Histologycal scores of ileal biopsies of wt and *Mif* ^−^/^−^ mice at 9 dpi. The horizontal line limits absence of inflammatory response (0 to 3) and necrosis (above 3). (D) Toxo DNA concentration was determined in ileal biopsies of wt and *Mif* ^−^/^−^ mice. One representative experiment out of three independent experiments. Data from 3 to 6 mice per group is represented by means ± SEM and p values determined by t-test (* p≤0.05, ** p≤0.01). Length of intestinal segments of control not infected wt and *Mif* ^−^/^−^ mice are represented by a dotted line in A. Cont, control not infected.

To determine the cause of reduced mortality of *Mif^−^/^−^* mice, we initially performed macroscopical and histopathological analysis of the small intestine of control and infected animals. At day 9 post infection, wt mice presented significant intestinal shortening compared to *Mif^−^/^−^* mice (Fig. 2A). This intestinal shrinkage observed in wt mice was associated with histological changes at the terminal ileum, including blunting of the villi, extensive necrosis and full thickness inflammatory infiltration of the lamina propria (Fig. 2B). Infected *Mif^−^/^−^* mice presented reduced necrosis and decreased loss of mucosal integrity, lamina propria and intestinal wall and more preserved areas of intestine. Tissue damage was determined by histopathological scores of the terminal ileum of infected mice, and most samples from wt mice presented higher scores when compared to *Mif^−^/^−^* mice (Fig. 2C). In fact, *Mif^−^/^−^* mice displayed less tissue damage in contrast to a significant increase in tissue parasitism (Fig. 2D). These findings indicate that MIF-mediated inflammatory response promotes tissue damage, contributing to morbidity and mortality, but also participates on *T. gondii* control at the intestinal mucosa.

### MIF regulates inflammatory cytokines in *T. gondii*-induced ileitis

**Figure 3:**
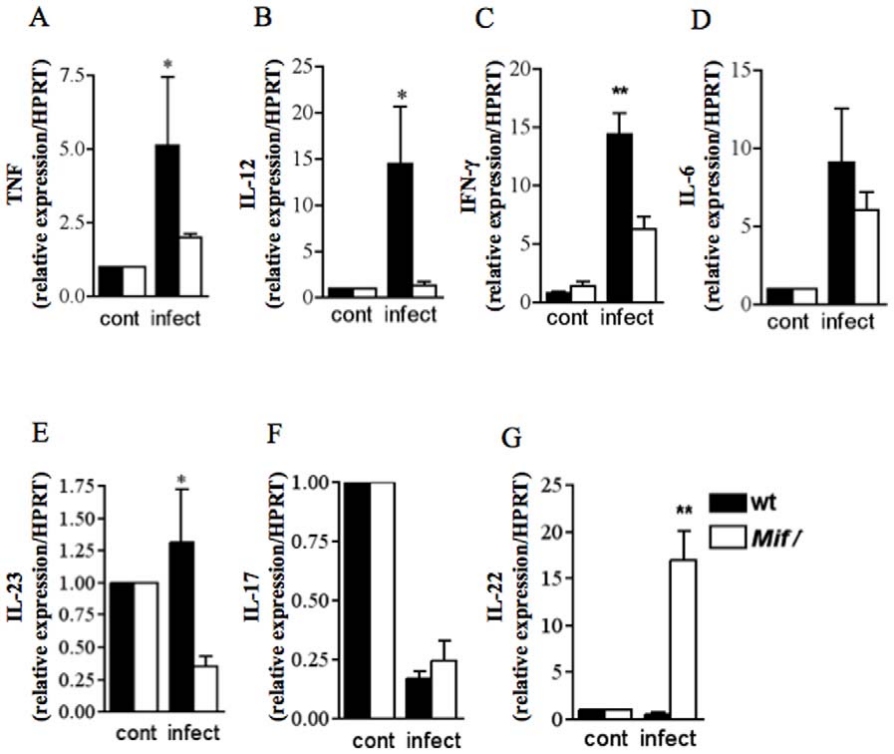
MIF upregulates proinflammatory responses in toxoplasmic ileitis. Real-time PCR of (A) TNF-α, (B) IL-12, (C) IFN-γ, (D) IL-6, (E) IL-23, (F) IL-17 and (G) IL-22 expression in ileal explants from control and infected wt and *Mif* ^−^/^−^ mice. Results are expressed as fold changes relative to HPRT mRNA expression. Data from 3 to 6 mice per group is represented by means ± SEM and p values determined by t-test (* p≤0.05, ** p≤0.01). Cont, control not infected.

**Figure 4:**
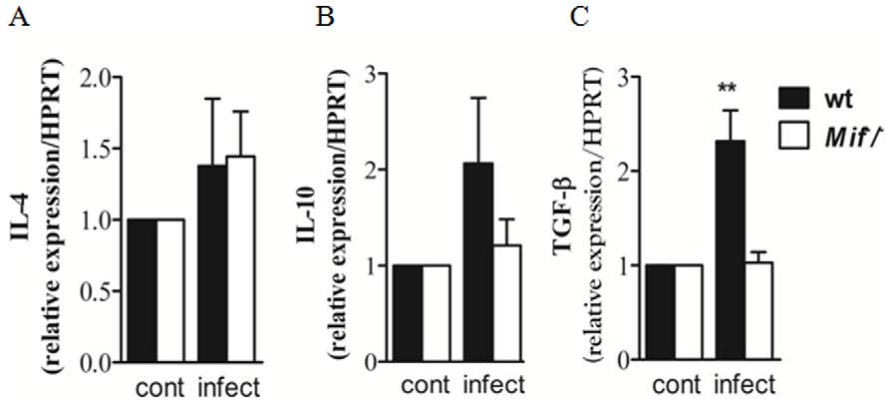
Reduced intestinal expression of IL-10 and TGF-β in *Mif^−/−^* mice. Quantitative RT-PCR of of (A) IL-4, (B) IL-10 and (C) TGF-β mRNA expression in ileal explants of control and infected wt and *Mif* ^−^/^−^ mice. Results are expressed as fold changes relative to HPRT mRNA expression. Data from 3 to 6 mice per group is represented by means ± SEM and p values determined by t-test (*p≤0.05). Cont, control not infected.

To investigate the mechanism by which MIF regulates the inflammatory response at the intestinal mucosa, we examined mRNA expression and the concentrations of inflammatory cytokines in the supernatants of ileal mucosal explants from wt and *Mif^−^/^−^* infected mice. TNF-α, IL-12, IFN-γ and IL-23 expression were decreased in infected *Mif^−^/^−^* mice when compared to wt mice (Fig. 3A–C, E). The mRNA expression of IL-6 and IL-17A were also similar in wt and *Mif^−^/^−^* mice (Fig. 3D, F). Surprisingly, IL-22 expression was significantly increased in infected *Mif^−^/^−^* compared to wt mice (Fig. 3G). To determine if IL-4, IL-10 and TGF-β could be implicated in the suppression of immunopathology and increased parasite burdens of *Mif^−^/^−^*, we examined the mRNA expression of these regulatory cytokines in the ileal mucosa explants of control and infected wt and *Mif^−^/^−^* mice. While IL-4 mRNA expression was similar in wt and *Mif^−^/^−^* mice at 9 days post infection (Fig. 4A), IL-10 mRNA expression and protein in ileal mucosa were reduced in *Mif^−^/^−^* compared to wt mice (Fig. 4B, data not shown). Similarly, *T. gondii* infected *Mif^−^/^−^* mice presented significant decrease of TGF-β expression when compared to infected wt mice (Fig. 4C). These results indicate that the reduced inflammatory response in the ileun of *Mif^−^/^−^* compared to wt mice is not due to up regulation of IL-4, IL-10 or TGF-β.

### *T. gondii* peroral infection induces decreased expression of MMP-9, but not MMP2, in the intestinal mucosa of *Mif* ^−^/^−^ mice

**Figure 5:**
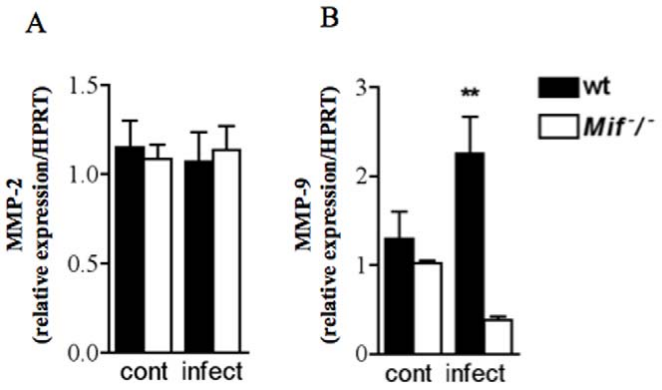
MIF upregulates MMP-9 but not MMP-2 in the terminal ileum of *T. gondii* infected mice. Quantitative real-time PCR of (A) MMP-2 and (B) MMP-9 mRNAs in ileal biopsies of control and infected (9 dpi) wt and *Mif* ^−^/^−^ mice. Results are expressed as fold changes to HPRT mRNA expression. One representative experiment out of two experiments is shown. Data of three to five mice per group are given as means ± SEM and p values were determined by Mann-Whitney (**p≤0.01). Cont, control not infected.

Matrix metalloproteinases have been implicated in the pathogenesis of IBD and most recently in *T. gondii*-induced ileitis [7]. Given that MIF regulates the expression of MMPs during pathological inflammatory responses such as arthritis [39], [40], we investigated the possible involvement of MMP-2 and MMP-9 in *T. gondii*-induced MIF-mediated ileitis. We determined the mRNA expression of MMP-2 and MMP-9 in the ileum of control and infected wt and *Mif^−^/^−^* mice. At day 9 post-infection, both control and infected wt and *Mif^−^/^−^* mice presented similar MMP-2 expression (Fig. 5A). However, MMP-9 was significantly reduced in the ileum of *Mif^−^/^−^* infected mice (Fig. 5B). These results suggest that MIF might contribute to pathological responses to *T. gondii* infection at the intestinal compartment via MMP-9 pathway.

### Systemic inflammatory responses are regulated by MIF and enhance *T. gondii*-induced pathology

**Figure 6:**
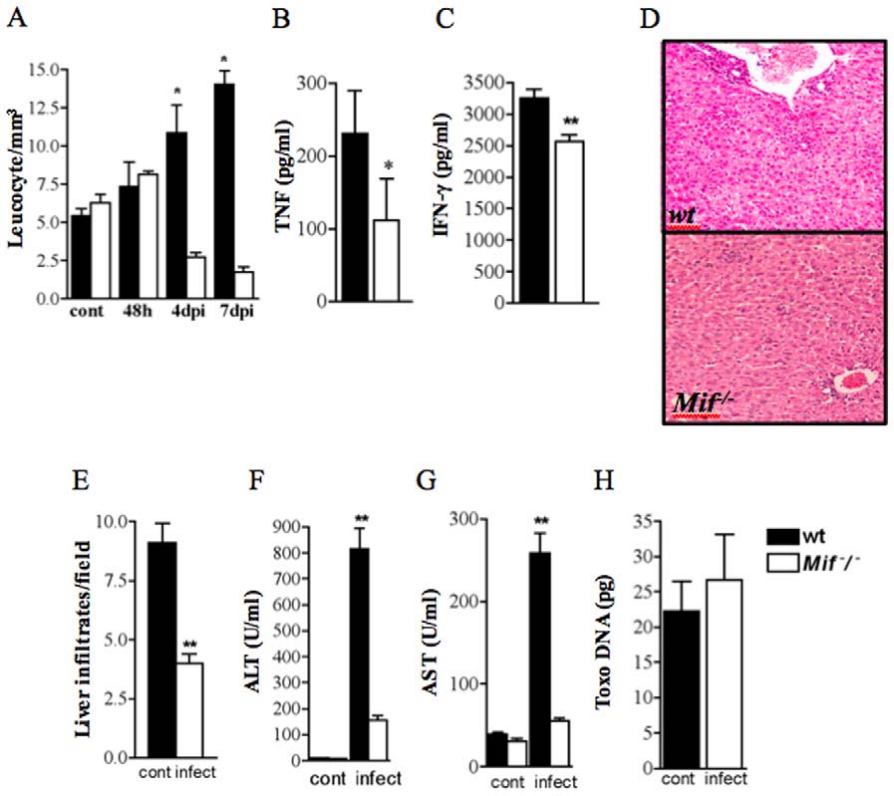
*T. gondii*-induced sepsis-like response is partially dependent of MIF. (A) Leukocyte counts were determined in peripheral blood at indicated times. (B) TNF-α and (C) IFN-γ concetrations in the plasma of wt and *Mif* ^−^/^−^ mice (n = 3–5) 9 dpi were measured by ELISA. (D) Histopathologycal analysis of the liver at 9 dpi from wt and *Mif* ^−^/^−^ mice. (E) Quantitative analysis of liver sections (3 slides/animal) of infected wt and *Mif* ^−^/^−^ mice mice was performed. Liver damage was also determined by quantifying serum concentrations of biochemical marker as (F) alanine aminotransferase (ALT), and (G) aspartate aminotransferase (AST). (H) Toxo DNA concentration was determined in the liver of wt and *Mif* ^−^/^−^ mice. One representative experiment out of six experiments is shown. Data of three to five mice/group are given as as means ± SEM and p values were determined by t-test (* p≤0.05, ** p≤0.01). Cont, control not infected.

The systemic inflammation during *T. gondii* infection resembles bacterial sepsis. Considering the critical role of MIF in the pathogenesis of experimental sepsis, we investigated several markers of systemic inflammation in *T. gondii*-infected wt and *Mif^−^/^−^* mice. Leukocyte counts were higher in wt mice at day 4 and 7 post-infection when compared to *Mif^−^/^−^* mice (Fig. 6A). Given that systemic inflammation during per oral infection are induced by pro-inflammatory mediators such as IFN-γ and TNF-α, we investigated the concentrations of these cytokines in the plasma of infected wt and *Mif^−^/^−^* mice. The concentrations of both cytikones were higher in wt compared to *Mif^−^/^−^* mice (Fig. 6B, C). Thrombosis was often associated with vascular inflammatory infiltration in the livers of wt and *Mif^−^/^−^* mice (Fig. 6D). Wt mice had increased numbers of mononuclear cell infiltrates in perivascular areas and scattered at the hepatic parenquima (Fig. 6D, E). In contrast, infected *Mif^−^/^−^* mice had preserved liver parenquima and reduced mononuclear cell infiltration (Fig. 6D, E). Wt mice presented higher alanine aminotransferase and aspartate aminotransferase plasma concentrations compared to *Mif^−^/^−^* mice, an indication of liver damage and dysfunction (Fig. 6F, G). Moreover, *T. gondii* DNA levels were similar in wt and *Mif^−^/^−^* mice (Fig. 6H). The findings suggest that *T.gondii*-induced systemic response is in part dependent on MIF, while parasite replication and dissemination is not affected by MIF.

## Discussion

In the present study we demonstrated that MIF increases the inflammatory response and tissue damage due to *T. gondii* oral infection in susceptible C57BL/6 mice. *Mif^−^/^−^* mice had reduced intestinal and systemic inflammation surviving more compared to wt mice, despite of an increase in intestinal parasite burden. This reduced inflammatory response in the intestine of *Mif^−^/^−^* mice was associated with decreased expression of inflammatory cytokines and MMP-9 comapared to wt mice. Despite of the lower expression of IL-23 compared to wt mice in ileal explants, IL-22 expression was siginificantly increased in *Mif^−^/^−^* mice. The originally local inflammatory response in the small intestine of *T. gondii*-infected wt mice had systemic repercussions similar to sepsis, including increased plasma concentrations of inflammatory cytokines, leukocytosis and liver damage, all less pronounced in *Mif^−^/^−^* mice. Together, these results indicate that MIF is involved in the response to *T. gondii* infection of the small intestine, increasing the ileitis and the systemic inflammatory response.

The peroral infection with *T. gondii* caused a severe ileitis characterized by intestinal shortening, extensive necrosis, blunting of the villi, loss of Peyer's patches and leukocyte infiltration in the lamina propria [1], [7], [41]. All these pathological parameters were significantly reduced on *Mif^−^/^−^* compared to wt mice. In natural *T. gondii* infection, gut-associated inflammatory response resembles that observed in Crohn's ileitis and it has been proposed as a model of IBD [1], [7], [41]. An essential role of MIF in experimental colonic inflammation has been demonstrated [20]. It has been also shown that MIF controls the production of inflammatory cytokines including TNF-α and IFN-γ involved in the pathogenesis of intestinal inflammation [42] In the present study, using a model of *T. gondii*-induced ileal inflammation, we demonstrated a critical role of MIF promoting the intestinal inflammatory response and the expression of TNF-α, IL-12 and IFN-γ.

A recent study showed that during peoral *T. gondii* infection IL-23 regulates the ileal inflammatory response independent of IL-17 [7]. We also observed that upon *T. gondii* infection IL-23 expression was upregulated in the intestine while IL-17 was reduced. The inflammatory role of IL-23 was dependent of increased IL-22 production that in turn promoted the ileal inflammation and tissue damage [7]. IL-22, a member of the IL-10 family, is important in epithelial cell homeostasis, in infection and inflammation [43]. In toxoplasmic ileitis, the inflammatory response is a result of IL-22 upregulation induced by IL-23 [7]. In fact, *T. gondii*-infected *IL-22^−^/^−^* mice have reduced intestinal inflammation and are more resistant than wt. Unexpectaly, we observed a significant increased of IL-22 expression in *Mif* ^−^/^−^ mice with reduction of IL-23 and TGF-β. Others have previously shown increased IL-22 production by T CD4+ lymphocytes in the absence of IL-23 [44], [45]. Thus our results suggest a previously unrecognized role of MIF inducing IL-23 and inhibiting IL-22 expression. Our results also implies a MIF-dependent pathogenic role of IL-22 in *T. gondii*-induced ileitis. Interestingly, an anti-inflammatory role of IL-22 in different models of colitis has been previously demonstrated [46]. Future studies will be importat to define the mechanism by which MIF controls IL-23 and IL-22 expression, and how MIF participates in the pathogenic role of IL-22 on *T. gondii*-induced intestinal inflammation.

We observed that MIF was essential to *T. gondii*-induced expression of MMP-9, suggesting that MIF could be involved in tissue remodeling/repair at the small intestine during *T. gondii* peroral infection by over expressing gelatinase B. Several studies have shown that MIF affects tissue remodeling and the expression of different metalloproteinases [40]. Previous studies demonstrated an increased expression and enzymatic activity of metalloproteinases in humans and mice with intestinal inflammation [7], [47]. MMP-2 and MMP-9 are elevated in the intestine of *T. gondii*-infected animals, however in these model, C57BL/6 mice lacking MMP-2 but not MMP-9 have reduced immunopathology of the small intestine [7]. Thus the exact role of the reduced expression of MMP-9 observed on *Mif^−^/^−^* upon *T. gondii* infection requires further investigations.

During intestinal parasitic infection, reduced anti-inflammatory responses could decrease parasite burden but intensify tissue damage, causing loss of epithelial barrier integrity and increased bacterial translocation [34]. Our results showed that MIF mediated *T. gondii*-induced ileitis does not affect IL-4 and increase IL-10 and TGF-β expression. The intestinal inflammatory response induced by *T. gondii* is complex and intestinal homeostasis requires reciprocal control of both Th1 and TGF-β mediated responses. Consequently, MIF exacerbation of intestinal inflammatory responses might involve not only increased pro-inflammatory responses but also TGF-β-mediated responses. MIF and TGF-β are involved in fibrotic responses in several conditions due to regulation of MMPs [48], [49]. Since both cytokines upregulate MMP-9 expression, it is possible that MIF regulates tissue remodeling in *T. gondii*-induced ileitis through a TGF-β/MMP-9 pathway.

The reduced inflammatory response and pathological damage in the small intestine of *Mif^−^/^−^* occurred in the context of higher *T. gondii* burden. It has been recently shown in a model of toxoplasmosis induced by intraperitoneal route that MIF is essentially protective [27]. In this model of systemic infection, *Mif^−^/^−^* had higher numbers of cysts in the brain. The increased susceptibility of *Mif^−^/^−^* mice systemically infected was related with reduced production of inflammatory cytokines, however these animals, in opposition to our findings, developed increased liver damage compared to wt mice [27]. *Mif^−^/^−^* mice of the Balb/c background are also more susceptible to *T. gondii* when infected by the oral route, presenting increased lethality and parasite burden in the brain and liver [28]. Compared to susceptible hosts such as C57BL/6, the infection of Balb/c mice with *T. gondii* by the oral route causes a less severe intestinal inflammatory response and a disease that tends to become chronic. These findings suggest that MIF-mediated *T. gondii*-induced pathological responses might be differently regulated depending on the route and site of infection, and the genetic background of the host.

We observed that *Mif^−^/^−^* mice had reduced signs of systemic inflammation compared to wt mice when infected by the oral route. The reduced systemic inflammation of *Mif^−^/^−^* mice was characterized by lower plasma concentrations of TNF-α and IFN-γ, reduced biochemical and histological parameters of liver damage and lower leukocytosis when compared to wt mice. Considering the pathogenic roles of TNF-α and IFN-γ in *T. gondii* oral infection, we postulate that enhancement of systemic inflammatory response is mediated by MIF acting in concert with TNF-α and IFN-γ [2], [4], [5]. A deleterious role of MIF was recently observed in a mouse model of lethal dengue virus infection, with reduced systemic inflammatory response in *Mif^−^/^−^* mice compare to wt mice [50]. Although the molecular mechanisms involved in toxoplasmic ileitis are not completely understood, new evidence indicates that co-factors such as Gram-negative intestinal microflora play a role in *T. gondii*-induced acute ileitis and systemic inflammation through LPS-induced TLR-4 signaling [51]. Since MIF up-regulates the expression of TLR-4 [17], it is possible that the reduced inflammatory response observed in *Mif^−^/^−^* mice upon *T. gondii* might be a result of reduced TLR-4 activation. Considering that MIF contributes to IBD even in the absence of TLR-4, it is possible that the effect of MIF on *T. gondii* pathogenesis occurs independently of its effects on TLR-4 expression. Interestingly, MIF also regulates the expression of TLR-11 [28], considered important in the recognition and response to *T. gondii* [52]. It has been suggest that the reduced expression of TLR-11 in the absence of MIF affects the maturation and activation of dendritic cells consequently hampering a protective immune response to *T. gondii* [28]. It will be important to characterize the role of TLR-4 and TLR-11 in the MIF-dependent small bowel injury caused by *T. gondii* in susceptible hosts.

In conclusion, we demonstrated that MIF participates in the pathogenesis of natural *T. gondii* infection in C57BL/6 mice, promoting an intense ileitis and a robust systemic inflammatory response that resulted in poor outcome during the acute phase. Similar to previous studies, we confirmed a central role of MIF in the control of parasite burden in different models of experimental toxoplasmosis [27], [28]. Although MIF has been regarded as essential in host protection during *T. gondii* infection, our findings demonstrated a pathogenic role of MIF in natural *T. gondii* infection in susceptible hosts by exacerbating IL-12, IFN-γ, TNF-α and IL-23 dependent responses. MIF also plays a role in tissue remodeling possibly by regulating TGF-β induced MMP-9. Thus, we propose that MIF actually exerts a bidirectional role in toxoplasmosis. This dichotomy is dependent on the route of infection and the genetic background of the host, and has been also observed with other pro-inflammatory cytokines [2]. In this scenario, MIF seems to compromise host protection by exacerbating intestinal tissue damage and by inducing a sepsis-like response. Previous studies demonstrating a participation of MIF on human *T. gondii* infection [27], [53], [54] enphasize the importance to understand the diffrential role of MIF on immunity and pathogenesis triggered by *T. gondii* in distinct tissues.
